# Molecular mapping of periodontal tissues using infrared microspectroscopy

**DOI:** 10.1186/1471-2342-5-2

**Published:** 2005-05-12

**Authors:** Allan Hynes, David A Scott, Angela Man, David L Singer, Michael G Sowa, Kan-Zhi Liu

**Affiliations:** 1 Dental Diagnostic and Surgical Sciences, University of Manitoba, Winnipeg, Canada; 2 Oral Health and Systemic Disease Research Group, University of Louisville School of Dentistry, Louisville, KY, USA; 3 Institute for Biodiagnostics, National Research Council, Winnipeg, Canada

## Abstract

**Background:**

Chronic periodontitis is an inflammatory disease of the supporting structures of the teeth. Infrared microspectroscopy has the potential to simultaneously monitor multiple disease markers, including cellular infiltration and collagen catabolism, and hence differentiate diseased and healthy tissues. Therefore, our aim was to establish an infrared microspectroscopy methodology with which to analyze and interpret molecular maps defining pathogenic processes in periodontal tissues.

**Methods:**

Specific key cellular and connective tissue components were identified by infrared microspectroscopy and using a chemical imaging method.

**Results:**

Higher densities of DNA, total protein and lipid were revealed in epithelial tissue, compared to the lower percentage of these components in connective tissue. Collagen-specific tissue mapping by infrared microspectroscopy revealed much higher levels of collagen deposition in the connective tissues compared to that in the epithelium, as would be expected. Thus inflammatory events such as cellular infiltration and collagen deposition and catabolism can be identified by infrared microspectroscopy.

**Conclusion:**

These results suggest that infrared microspectroscopy may represent a simple, reagent-free, multi-dimensional tool with which to examine periodontal disease etiology using entirely unprocessed tissue sections.

## Background

Periodontitis is defined by the inflammatory destruction of the supporting structures of teeth, including the periodontal ligament and alveolar bone. Periodontal diseases are generally chronic in nature and usually persist in the absence of treatment [1,2]. These diseases are the result of exposure of the periodontium to dental plaque biofilms that accumulate on the teeth to form bacterial masses at or below the gingival margin [3].

**Figure 1:**
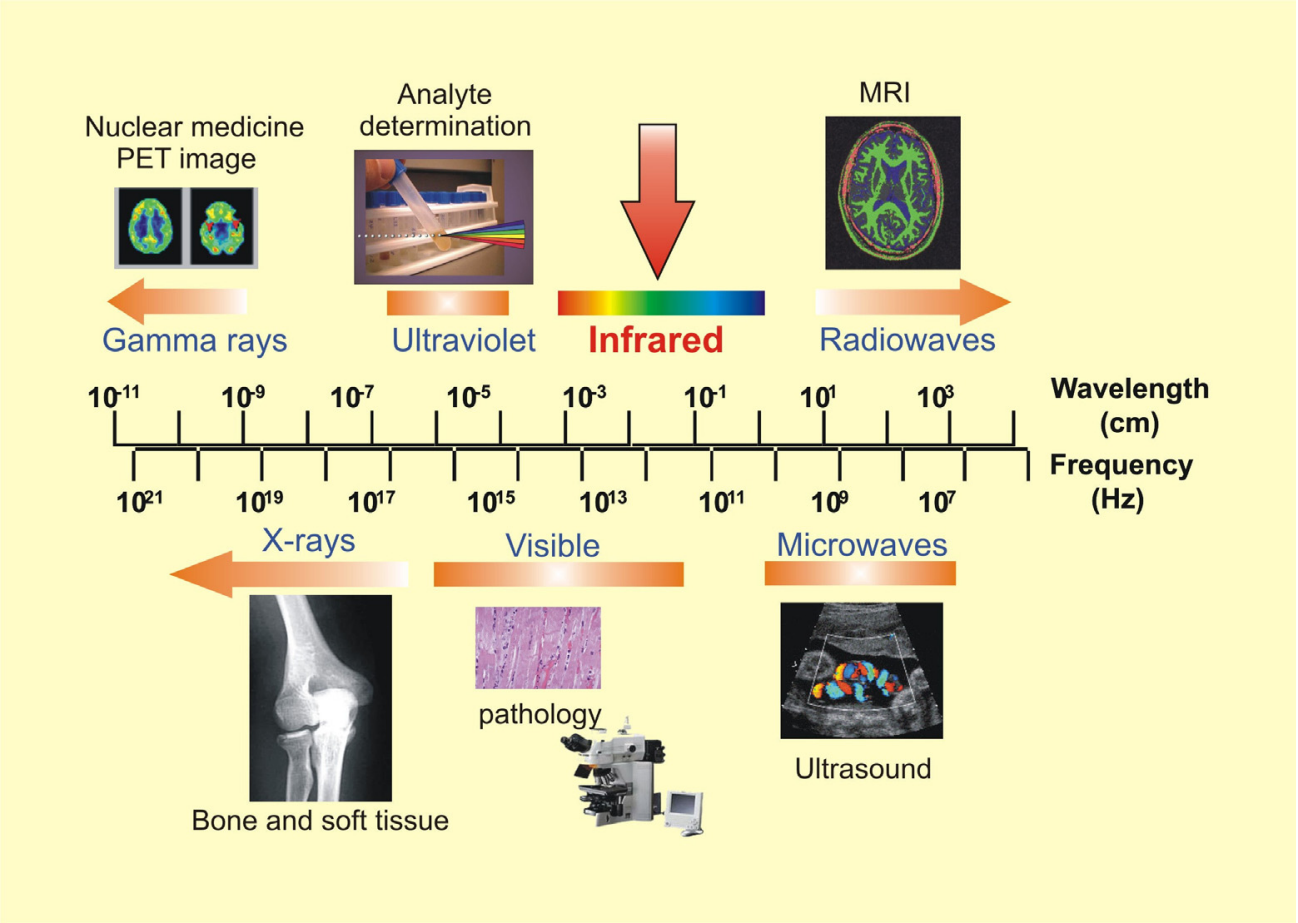
**Biomedical applications of the electromagnetic spectrum**. The classic electromagnetic spectrum is shown aligned with common, established biomedical applications. The infrared region of the electromagnetic spectrum lies between the visible and microwave regions, as indicated by the red arrow.

Biomedical utilization of the electromagnetic spectrum of light has revolutionized the practice of medicine over the centuries, most recently through the dramatic healthcare advances afforded by the development of magnetic resonance imaging (see Figure 1). The last region of the spectrum to be applied to the practice of medicine is the infrared region. Infrared (IR) spectroscopy is now being increasingly utilized in multiple biomedical settings [4].

**Figure 2:**
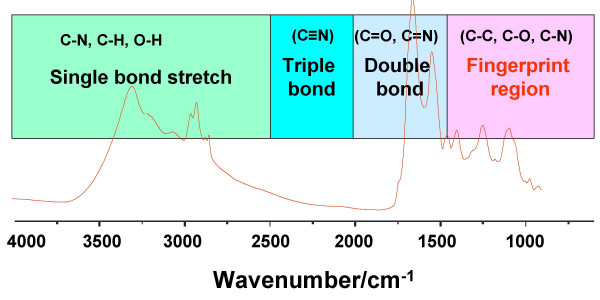
**Representative spectrum in the mid-IR region**. The region below 1500 cm^-1 ^is defined as the fingerprint region. The region above 1500 cm^-1 ^is the functional group region.

IR spectroscopy can distinguish differences in the characteristics of diverse molecules by probing chemical bond vibrations and use these molecular and sub-molecular profiles to define and differentiate "diseased" and "healthy" tissues [4]. As covalent bonds vibrate, they absorb energy in the form of IR light (see Figure 2). The wavelength of light that is absorbed depends on the nature of the covalent bond (e.g. C=O, N-H), the type of vibration (bending, stretching, etc.), and the environment of the bond. The IR spectrum of a tissue sample can be regarded as molecular fingerprint of the tissue. If this molecular fingerprint is modified by a disease process, then IR spectroscopy can be used to detect and monitor the disease process.

IR microspectroscopy is a relatively new technique in which infrared spectra are observed through a microscope that transmits and detects infrared radiation. IR microspectroscopy has been previously utilized to monitor variations in the catabolism and anabolism of collagen and other components within cardiac tissues and oral cancer [5,6]. Collagens exhibit a series of unique IR absorption bands between 1000 and 1300 cm^-1^. Specifically, the strong band at 1204 cm^-1 ^has been identified as typical of collagen deposition [5,6]. By integrating the intensity of this absorption band, one can readily plot molecular contour maps that clearly delineate areas of collagen deposition. Inflammation-driven collagen degradation is a hallmark of periodontitis [7,8], with strong dissolution of collagen types I and III observed [9,10].

Particular absorptions are assigned to various functional groups in an attempt to extract biochemical information. Absorptions between 1620–1680 cm^-1 ^are usually attributed to amide I vibration of proteins, while absorptions at 1080 and 1240 cm^-1 ^are attributed to PO_2_^- ^symmetric and asymmetric stretching vibrations of DNA phosphodiester groups [4]. Using this data qualitative and semi-quantitative information can be extracted from the spectra.

Therefore, we set out to establish an IR microspectroscopic methodology that would allow the analysis and interpretation of molecular maps defining pathogenic processes in unprocessed periodontal tissues. This task is much more difficult in periodontal tissues in comparison to most other tissues due to the following factors; (1) Periodontal soft tissues are very thin (to a few mm); (2) Periodontal tissues excised at surgery are generally small (around 5 mm^2^); and (3) Excised periodontal tissues are particularly friable. Each of these factors present problems in embedding, sectioning, and orientating unfixed periodontal samples.

## Methods

### Subjects

Patients referred to the Graduate Periodontics Clinic, University of Manitoba for periodontal therapy and giving written, informed consent were recruited. Inclusion criteria were a clinical diagnosis of chronic periodontitis and a requirement for periodontal surgery at a diseased site (probing pocket depth ≥ 5 mm, bleeding on probing, and clinical attachment loss ≥ 3 mm). Exclusion criteria were tobacco smoking, pregnancy, a requirement for antibiotic prophylaxis prior to periodontal probing, prolonged anti-inflammatory medications within the past 3-months (e.g. NSAIDs, steroids, antibiotics, or immunosuppressants), any systemic condition that may interfere with the study, such as inflammatory diseases, diabetes or blood dyscrasias, and lesions of the gingiva unrelated to plaque-induced periodontal disease. All subjects had received oral hygiene instruction and root planing prior to surgery.

### Expired-air Carbon Monoxide measurement

Self-reported non-smoking status was validated at the time of recruitment by analysis of expired-air CO concentrations (PiCO meter, Bedfont Sci., UK and associated PiCO chart software), calibrated according to the manufacturers instructions. Non-smokers were required to exhibit expired-air CO concentrations <10 ppm.

### Periodontal tissue collection and processing

On the day of surgery, prior to any anesthesia being administered, clinical measurements were obtained from the surgical site: probing depths, bleeding on probing and clinical attachment level (in that order). Twenty periodontal tissue samples were obtained from patients in good general health who were undergoing surgical treatment for chronic periodontitis. Mid-interproximal tissue was preferred, as these sites show increased clinical and histological signs of inflammation compared to other gingival sites [11]. Periodontal tissue samples were snap frozen in isopentane supercooled in liquid nitrogen and then stored at -80°C.

A small amount of optimal cutting tool embedding media (Miles Inc., Elkhart, IN.) was applied to the tissue samples in order to facilitate attachment to the cryotome. A series of 10 μm sections were then cut, mounted onto an IR transparent barium fluoride window, and air-dried. Adjacent 10 μm samples were mounted and processed for conventional histology including visualization of tissue integrity and inflammatory foci by H & E staining.

### Infrared microspectroscopy

Infrared microspectroscopy was performed using a Bruker FTIR spectrometer and IR microscope system equipped with a liquid nitrogen cooled mercury cadmium telluride detector. For IR spectral acquisition, the microscope aperture was closed to allow the IR beam to illuminate an area of tissue measuring 50 μm × 50 μm, thereby masking all other regions of the tissue section. For each 50 μm × 50 μm demarcation, 64 interferograms were collected. The signal was averaged against a blank area as background and Fourier-transformed to generate IR spectra with a nominal resolution of 4 cm^-1^. After each acquisition, the stage was stepped 50 μm under computer control and the next spectrum acquired. This process was repeated until the complete area of interest was mapped. General biochemical mappings regarding the specific components of proteins, lipid, and DNA were obtained using a chemical imaging method. Specifically, the bands used for protein mapping were the amide I band (1654 cm^-1^); lipid mapping used the lipid CH stretching vibrations at 2800–3000 cm^-1^; and DNA mapping employed the PO_2_^- ^symmetric stretching vibration of DNA phosphodiester groups at 1080 cm^-1^. To highlight collagen deposition, a unique collagen IR band at 1204 cm^-1 ^was used to generate tissue maps, as we have described previously in cardiac tissues [5]. A digital CCD video camera was coordinated to the IR microscope to record each mapped area for future band and area correlation. Microphotography of the identical mapping position in the adjacent H and E-stained tissue section allowed the comparison of general histological features with the IR mapping data.

### Data processing for IR microspectroscopy

Because most IR bands are broad and are composed of overlapping components, it is necessary to pre-process the original spectra by applying a band-narrowing algorithm that separates the individual bands. All data processing was performed using the Cytospec V software package . To permit a useful comparison of the cluster analysis, uniformly pre-treated data was used. All the original IR spectra were converted into second derivative spectra using the Savitzky/Golay algorithm with a 9-point window for the multivariate statistical analysis. Derivative spectra were scaled before the cluster analysis where the sum squared deviation over the indicated wavelengths equals unity (vector normalization) [12]. The unsupervised cluster analysis used Ward's minimum variance algorithm and Euclidean distances as distance measure. The Euclidean distance between spectra is calculated and the pair of spectra with the least distance is grouped to create a cluster. Then the separation between this cluster and all other spectra is calculated and another two closest spectra/clusters are joined to form a new cluster. This procedure continues until all spectra/clusters are combined. In this unsupervised classification no information about the disease state of the samples is needed, only the similarity or dissimilarity of their infrared spectra is used for this classification.

## Results

**Figure 3:**
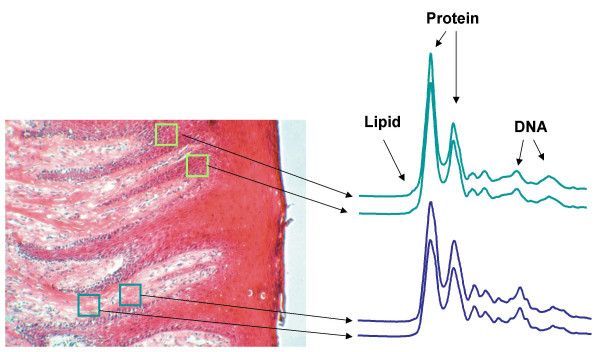
**Comparison of periodontal tissues visualized by IR microspectroscopic mapping and by conventional H and E staining**. Major periodontal tissue components (DNA, protein, and lipid), as determined by IR microspectroscopic mapping, are correlated with conventional H&E staining in a representative periodontal tissue section. IR spectra obtained from within the square boxes at different areas of the tissue section (periodontal epithelium and nearby connective tissue) are shown.

IR microspectroscopic imaging generated molecular tissue maps that provide a spectral signature of the intensity and spatial location of the chemical components of the diseased periodontal tissues examined. A representative microphotograph of periodontal tissue stained with H and E is shown in the left panel of Figure 3, while the spectra generated from defined area by IR microspectroscopy indicated by the square box are shown in the right panel. The H and E staining reveals typical histological constitution of periodontal tissue, i.e. the gingival epithelium comprising the epithelial layer that covers the external surface of the gingival and underlying connective tissue composed of gingival fibers, ground substance, and cells, including neural and vascular elements. Spectra from both the epithelium and connective tissues display the distinctive infrared spectral signatures of specific molecular structures, such as DNA, protein and lipids, as shown in the right panel.

**Figure 4:**
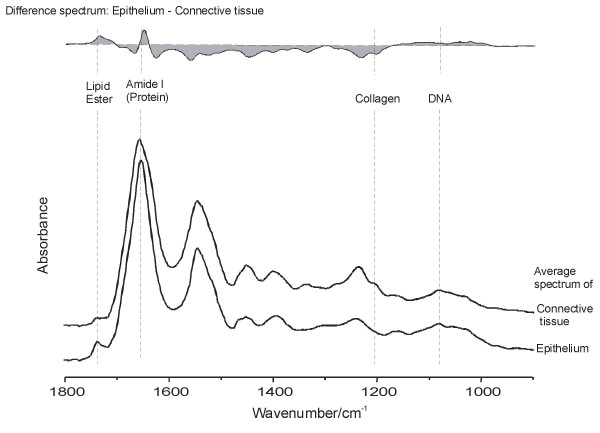
Comparison of the mean infrared absorption spectra* of epithelial (lower spectrum) and connective tissue (upper spectrum) and the epithelial-connective tissue difference spectrum. *Spectra are baseline corrected.

**Figure 5:**
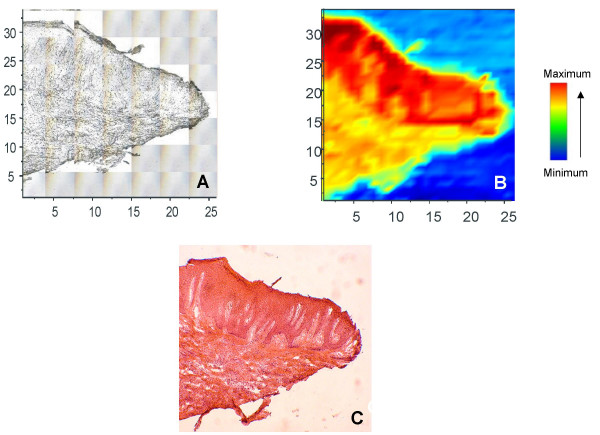
**Comparison of unstained periodontal tissue section with H and E staining and the associated IR mapping**. The original periodontal tissue used for IR mapping (A), as defined by the adjacent tissue stained with H&E (B), correlates very well with the IR chemical map based on protein band intensity (C). The color bar indicates the relative intensity of the specific IR band in the tissue.

In order to reveal more subtle molecular differences in the periodontal tissue, the average spectra of these areas were generated using cluster analysis, as shown in Figure 4. As expected, both spectra show the signature of typical tissue components, i.e. total protein and collagen-specific signals, lipids (membranes), and nucleic acids. For instance, the absorptions at 1654 cm^-1 ^arise from the amide I C=O stretching vibrations of the peptide groups in proteins [13]. The band at 1080 cm^-1 ^is due to vibration of the phosphodiester groups in DNA that can be used to identify the content of cellular nucleus. The information regarding relative lipid concentration in both tissues can be found at the 1740 cm^-1 ^band that originates from the ester C=O group while collagen substance in the tissue can be traced by looking at the specific band at 1204 cm^-1^, as marked in the figure [5]. The top panel in Figure 4 is the difference spectrum, generated by subtracting the average spectrum of connective tissue from that of epithelium. The difference spectra help identify the specific molecular components that differ most between the two groups of spectra. Upwardly pointing peaks (positively shaded areas) are indicative of the presence of more of a certain molecular component in the epithelium tissue compared to that in the connective tissue, and vice-versa for the downwardly pointing peaks (negatively shaded areas). There are several important changes in the IR spectra that involve molecular vibrations of proteins, including collagen, lipids, and DNA. As expected, a higher cellular constituent content (protein, lipids and DNA) was found in the epithelial tissue, compared to the lower percentage of these components in the connective tissue. However, the collagen content was much higher in the connective tissue than in the epithelium.

**Figure 6:**
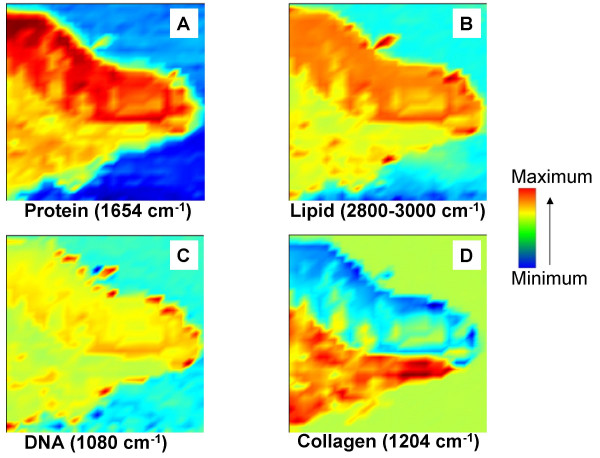
**IR chemical mappings in periodontal tissue**. Four major molecular distributions (total protein; lipid; DNA; collagen) in periodontal tissue (A-D) were generated based on the corresponding IR molecular signals. Higher concentrations of the major cellular components were observed in the periodontal epithelium (A-C) whereas higher amounts of collagen exist in the connective tissue (D), as expected.

Based upon these molecular differences in periodontal tissues, IR chemical mappings were established and compared with conventional H and E-stained sections, as shown in Figure 5. Specifically, regions of interest in the periodontal tissue were identified using the H and E stained section and a high quality microscope in the visible range. Then, the corresponding features were identified by anatomical landmarks on the unstained adjacent tissue sections; subsequently, the tissue section was transferred to the IR microscope and aligned using the anatomical landmarks, as shown in Figure 5A and 5B. The numbers in the *X*- and *Y*-axes indicate the step scans employed in the acquisition of IR mapping. Therefore, a total of 962 spectra in this section were obtained and stored in the hypercube. To visualize the spectral data from the hypercube, the easiest way is to present "horizontal" slices through the hypercube, which displays intensity values at a given wavenumber for all spectral vectors as a false color representation [14]. The intensity value of each spectrum is assigned a color code and displayed against the *X *and *Y *coordinates of the spectral element. By examining different "color slices" (i.e., the intensities at different spectral elements), variations in the chemical composition can be detected for various pixels in the hypercube. As demonstrated in Figure 5C, the chemical IR mapping based on protein concentrations in the periodontal tissue revealed highest protein content in the epithelium, which was well correlated with tissue components observed by conventional H and E staining. The cell density in the epithelial tissue is significantly higher than that in the connective tissue (Figure 3). The epithelial cells provide the major source of protein noted in the IR spectra, especially at the level of functional group mapping. However, although the epithelial tissue contains more total protein than the connective tissue, the latter contains much more collagen. Other important cellular chemical mappings were also generated from the IR maps, as shown in Figure 6A–D. These false color IR molecular have the familiar appearance of traditional histological sections.

**Figure 7:**
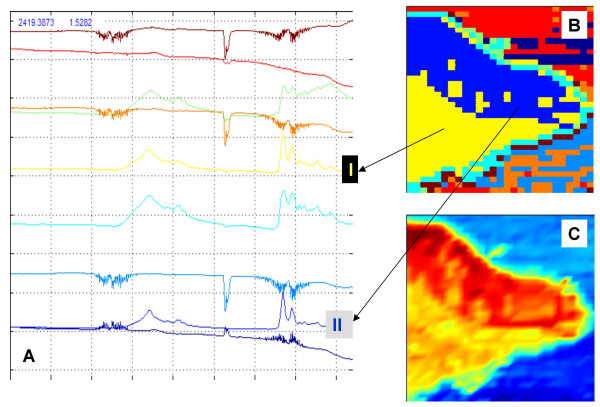
**Cluster mapping of periodontal tissue**. Average spectra were generated from nine groups based on their overall spectral features (A); each representing one class in the cluster map of periodontal tissue (B) produced by the cluster analysis. Good correlation can be observed between the cluster mapping (B) and corresponding chemical mapping (protein) (C) based on the differential spectral features of the epithelial and connective tissues.

Finally, we employed a multivariate statistical method, CLA (cluster analysis), to verify that the epithelial cells can be readily separated from connective tissues, as shown in Figure 7. The advantage of this computational approach is that it operates unsupervised: no prior knowledge of reference spectra of any tissue type is required, and characteristic spectra and membership of the families of spectra identified by the characteristic spectra are established, regardless of the number of different spectral families and the differences between spectral families [14]. This cluster analysis uses a large portion of the spectrum, rather than a few selected points, regions, or integrated regions, to determine whether spectra are related or not. Because the differences between spectra of normal and diseased tissue are generally small, this method offers the advantage of emphasizing differences in the overall shape, rather than selected intensities, for the discrimination of spectra. In this study, the entire range from 900–1800 cm^-1 ^was used and nine groups based on their spectral features were generated.

In the final step of cluster analysis, all spectra in the same family are assigned a color code, and small, colored squares are drawn at the pixel coordinates of all spectra belonging to the same family to produce our false color maps. As shown in Figure 7B, the blue color (line II) indicates that the spectra from this group were in the same family, originating from the epithelium, like the IR protein mapping of the same tissue shown in Figure 7C. The yellow color in Figure 7B (line I) represents those similar spectra generated from connective tissue. Panel A in Figure 7 displays the average spectrum produced by the cluster analysis with color codes. In other words, the average spectrum from epithelium and connective tissues are in blue and yellow, marked II and I, respectively, and also correspond to the spectra analyzed in full detail in Figure 4.

## Discussion

IR microspectroscopy possesses numerous advantages over traditional approaches to pathology. Fixation and staining of tissues are not required before histological viewing, little or no sample preparation is necessary and only minimal technical expertise is required by the operator. The method lends itself readily to rapid, high-volume repetitive measurements. IR microspectroscopy is non-destructive, meaning that the sample may be saved and passed on for further measurements if required. Furthermore, IR microspectroscopy provides information concerning the molecular structure of the tissue and multiple analytes may be measured simultaneously from a single spectrum. This combination of features is simply unavailable from visual microscopy.

Our initial data suggest that infrared microspectroscopy represents a suitable tool with which to simultaneously monitor multiple disease markers in periodontal biopsies, including cellular infiltration, collagen catabolism, and other differences in the molecular profile of diseased tissues. We have established a methodology by which IR microspectroscopy is capable of revealing several major biochemical components and specific features, including collagen content, in the studied tissue using "digital staining", without the need for any chemical reagents or probes. IR maps of inflammation-driven collagen degradation in periodontal tissue sections can therefore be constructed and analyzed. The promising preliminary results, obtained in establishing this IR microspectroscopic methodology, suggest a potential role for infrared microspectroscopy in understanding the inflammatory processes underlying the progression of periodontitis.

The major problem in adapting IR technology to the study of inflammatory processes in gingival tissues was the small size and fragility of the periodontal biopsies themselves. However, we are able to process these small tissue samples and can clearly differentiate predominantly cellular and predominantly acellular areas of tissue, and visualize areas of collagen deposition and degradation. The next steps will be to compare IR microscopic maps of diseased and healthy tissues; to correlate NIR-based definitions of pathological tissue changes with disease severity; and to correlate IR maps with classical clinical signs of periodontal diseases, such as edema, gingival bleeding, and periodontal pocket depths on probing, and clinical attachment loss.

## Conclusion

As inflammatory cell infiltration and subsequent collagen degradation are hallmarks of periodontitis [7-10], and because IR spectral analyses can determine such tissue events, in addition to multiple other tissue changes at the molecular and sub-molecular level, then this IR microspectroscopic methodology can now be applied to hypothesis-driven research that aims to identify disease-related pathological changes in periodontal tissues.

## List of abbreviations

FTIR, Fourier-transform infrared; IR, infrared

## Competing interests

The authors have no conflicting interests or financial implications related to the publication of this review article. The research activities described herein are humanitarian (non-profit making) in nature. However, certain application of infrared technology may be patentable.

## Authors' contributions

The original hypothesis was developed by DAS, K-ZL, and DLS. DAS performed the literature review included in the manuscript. AGH and DLS identified suitable clinical cases. AGH performed the surgeries and prepared tissue sections for sectioning. AGH and AM performed most of the infrared microspectroscopy experiments. K-ZL, DAS and MGS performed the extrapolation of data from the IR spectra and the spectral data analysis. All authors made editorial contributions to the manuscript.

## Pre-publication history

The pre-publication history for this paper can be accessed here:
